# Importance of Gradients in Membrane Properties and Electrical Coupling in Sinoatrial Node Pacing

**DOI:** 10.1371/journal.pone.0094565

**Published:** 2014-04-23

**Authors:** Shin Inada, Henggui Zhang, James O. Tellez, Nitaro Shibata, Kazuo Nakazawa, Kaichiro Kamiya, Itsuo Kodama, Kazuyuki Mitsui, Halina Dobrzynski, Mark R. Boyett, Haruo Honjo

**Affiliations:** 1 National Cerebral and Cardiovascular Center Research Institute, Suita, Osaka, Japan; 2 Institute of Cardiovascular Sciences and Biological Physics Group, University of Manchester, Manchester, United Kingdom; 3 Shinjuku Mitsui Building Clinic, Tokyo, Japan; 4 Research Institute of Environmental Medicine, Nagoya University, Nagoya, Japan; 5 Graduate School of Advanced Science and Technology, Tokyo Denki University, Tokyo, Japan; Gent University, Belgium

## Abstract

The sinoatrial node (SAN) is heterogeneous in terms of cell size, ion channels, current densities, connexins and electrical coupling. For example, Na_v_1.5 (responsible for *I*_Na_) and Cx43 (responsible for electrical coupling) are absent from the centre of the SAN (normally the leading pacemaker site), but present in the periphery (at SAN-atrial muscle junction). To test whether the heterogeneity is important for the functioning of the SAN, one- and two-dimensional models of the SAN and surrounding atrial muscle were created. Normal functioning of the SAN (in terms of cycle length, position of leading pacemaker site, conduction times, activation and repolarization sequences and space constants) was observed when, from the centre to the periphery, (i) cell characteristics (cell size and ionic current densities) were changed in a gradient fashion from a central-type (lacking *I*_Na_) to a peripheral-type (possessing *I*_Na_) and (ii) coupling conductance was increased in a gradient fashion. We conclude that the heterogeneous nature of the node is important for its normal functioning. The presence of Na_v_1.5 and Cx43 in the periphery may be essential for the node to be able to drive the atrial muscle: Na_v_1.5 provides the necessary depolarizing current and Cx43 delivers it to the atrial muscle.

## Introduction

Joyner and van Capelle [1] have argued that it is theoretically difficult for the sinoatrial node (SAN), the pacemaker of the heart, to drive the surrounding atrial muscle, which is non-pacemaking and more hyperpolarized than the SAN. They suggested that the solution is a gradient in electrical coupling between the centre of the SAN and the surrounding atrial muscle [1]. However, more recently, our work has highlighted the importance of Na^+^ current (*I*_Na_) in the periphery of the SAN for the SAN to be able to drive the atrial muscle [2] and the purpose of this study was to systematically investigate these two factors (electrical coupling and *I*_Na_) in SAN function.

**Figure 1:**
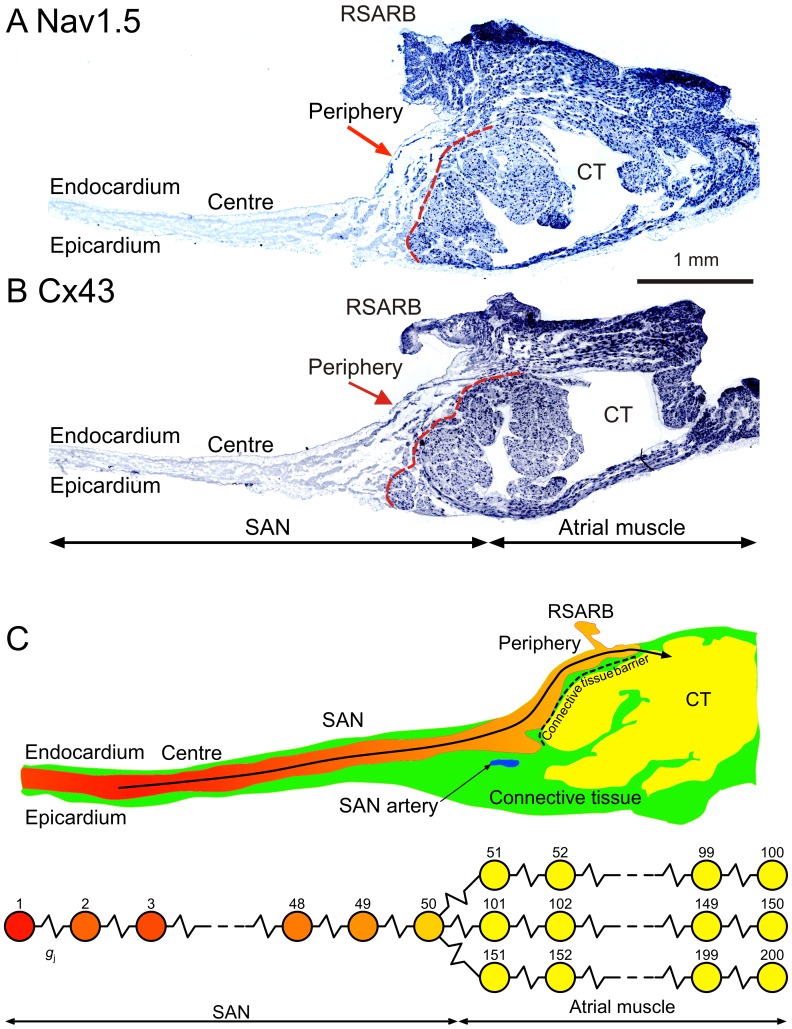
‘Real’ SAN (rabbit) and 1D model of SAN. **A**, expression of Na_v_1.5 mRNA in section cut perpendicular to crista terminalis through intercaval region and crista terminalis. **B**, expression of Cx43 mRNA in another section through intercaval region and crista terminalis. **C**, schematic diagram of section through intercaval region and crista terminalis. *Red*, central SAN tissue; *orange*, peripheral SAN tissue; *yellow*, atrial muscle; *green,* connective and fatty tissue. Arrow shows conduction pathway from leading pacemaker site in centre of SAN through periphery of SAN and into atrial muscle of crista terminalis. 1D model from centre of SAN to atrial muscle via periphery of SAN is shown at bottom. SAN, sinoatrial node. CT, crista terminalis. RSARB, right branch of sinoatrial ring bundle.

The SAN is a complex structure. In the rabbit, the majority of the nodal tissue is located in the thin intercaval region (between the superior and inferior vena cava). This is the *centre* of the SAN and it is normally the leading pacemaker site. The SAN tissue continues from the intercaval region onto the crista terminalis (a thick bundle of atrial muscle; Fig. 1). SAN tissue is mainly located on the endocardial surface of the crista terminalis and is the *periphery* of the SAN (Fig. 1). The periphery of the SAN is normally the conduction pathway for the action potential from the leading pacemaker site in the centre to the atrial muscle, although the periphery of the SAN has intrinsic pacemaker activity and it can act as the leading pacemaker site in some circumstances [3]. It has been suggested that the periphery of the SAN, except at its furthest extent, is separated from the atrial muscle of the crista terminalis by connective tissue (red dashed lines; Fig. 1A, B) [3]. The furthest extent of the rabbit SAN is marked by the right branch of the sinoatrial ring bundle (RSARB, a vestige of the embryonic venous valve; Fig. 1) – a ball-like tissue preparation from this region shows vigorous pacemaker activity [3].

The SAN is not uniform and there are characteristic differences between the centre and periphery [3]. In the centre of the rabbit SAN, the cells are small (length, ∼63 µm; calculated cell capacitance, *C*_m_, ∼40 pF) [4], the upstroke of the action potential is slow (maximum upstroke velocity, d*V*_m_/d*t*_max_, ∼2 V/s), the action potential is long, the maximum diastolic potential (MDP) is relatively positive (∼−60 mV) and the intrinsic pacemaker activity is paradoxically slow [3]. In contrast, in the periphery of the rabbit SAN, the cells are larger (length, ∼101 µm; calculated *C*_m_, ∼64 pF) [4], the action potential upstroke is faster (d*V*_m_/d*t*_max_, ∼50 V/s), the action potential is shorter, the MDP is more negative (∼−75 mV) and the intrinsic pacemaker activity is faster [3]. The regional differences in electrical activity in the rabbit have been observed in both the intact SAN and small balls of nodal tissue (∼0.3 mm in diameter) prepared from the different regions of the SAN. According to the gradient model of the SAN, the regional differences in active membrane properties are the result of regional differences in ionic current densities in the nodal cells [3]. Evidence for this has come from the study of isolated rabbit SAN cells of different sizes [3]. As already explained, in the centre of the SAN the cells are small, whereas in the periphery the cells are large - small cells (with a *C*_m_ of ∼20 pF) isolated from the SAN generally have the electrophysiological properties of the centre of the SAN, e.g. d*V*_m_/d*t*_max_ of ∼2 V/s and MDP of ∼−50 mV, whereas large cells (with a *C*_m_ of ∼60 pF) isolated from the SAN generally have the electrophysiological properties of the periphery of the SAN, e.g. d*V*_m_/d*t*_max_ of ∼50 V/s and MDP of ∼−65 mV [3], [5]. Ionic current densities vary between small and large cells in a manner that explains the different electrophysiological properties of the small and large cells (and the centre and periphery of the SAN) [5], [6]. For example, small cells (*C*_m_ of ∼20 pF) lack *I*_Na_, which explains the slow action potential upstroke of small cells (and also the centre of the SAN) [3]. In contrast, large cells (*C*_m_ of ∼60 pF) have a large *I*_Na_, which explains the fast action potential upstroke of large cells (and also the periphery of the SAN) [3]. Interestingly, an almost identical relationship between cell size and electrophysiological properties has been observed in rabbit atrioventricular node cells [7]. Na_v_1.5 is responsible for *I*_Na_ and Na_v_1.5 mRNA is distributed in the expected manner in the rabbit SAN [8], [9] – Na_v_1.5 mRNA is abundantly expressed in the atrial muscle, but is absent from the centre of the SAN (Fig. 1A). Na_v_1.5 mRNA is also abundantly expressed in the RSARB (Fig. 1A). This explains why *I*_Na_ is present and the action potential upstroke is fast in the periphery of the SAN (as well as the atrial muscle), but *I*_Na_ is absent and the action potential upstroke is slow in the centre.

Within the rabbit SAN, there are also regional differences in gap junctions responsible for electrical coupling [9], [10]. It is well known that electrical coupling is poor in the centre of the rabbit SAN – the coupling conductance between SAN cell pairs is 0.6–25 nS [11] (as compared to 30–635 nS between rabbit atrial cell pairs [12]), the space constant is low [3] and the conduction velocity is low (∼2 cm/s as compared to ∼70 cm/s in rabbit atrial muscle) [3]. The principal connexin (Cx) isoform responsible for gap junctions in the heart is Cx43. Cx43 forms medium conductance (60–100 pS) gap junctions and is abundantly expressed in the working myocardium including the atrial muscle. In addition, Cx40, a high conductance Cx isoform, is also expressed and plays an important role in cell-to-cell coupling in the atrial muscle. Consistent with the poor electrical coupling in the centre of the SAN, Cx43 is not expressed in the centre of the SAN [3] – Cx43 mRNA is abundantly expressed in the atrial muscle, but is absent from the centre of the rabbit SAN (Fig. 1B). In the centre of the rabbit SAN, gap junctions are formed from Cx45, a low conductance (20–40 pS) connexin isoform [8], [9]. In contrast to the centre of the rabbit SAN, electrical coupling is likely to be better in the periphery, because gap junctions are formed from Cx43 as well as Cx45 in the periphery [3] – Cx43 mRNA is also expressed in the rabbit RSARB (Fig. 1B). Consistent with this, in the rabbit SAN, the conduction velocity is higher in the periphery (∼30 cm/s) than in the centre (∼2 cm/s) [3].

The physiological importance of the heterogeneity in active membrane properties and electrical coupling in the SAN is not fully understood and, in the present study, we investigated the effects of the regional differences in the SAN on the initiation and propagation of the action potential in one-dimensional (1D) and two-dimensional (2D) models of the SAN and surrounding atrial muscle.

## Methods

### 1D Model of SAN and Surrounding Atrial Muscle

In the rabbit SAN, the leading pacemaker site is usually located in the centre of the SAN in the intercaval region 0.5–1.0 mm from the border of the crista terminalis, and the action potential propagates from the leading pacemaker site to the periphery of the SAN and then into the atrial muscle of the crista terminalis (Fig. 1C). We represented this conduction pathway as a 1D string of cells. This consisted of a string of 50 SAN cells (varying from the central- to the peripheral-type) and three parallel strings of 50 atrial cells (Fig. 1C bottom). We used the model of Kurata et al. [13] to compute the action potential in SAN cells and the model of Lindblad et al. [14] to compute the action potential in atrial cells (Kurata-Lindblad model).

In the centre of the SAN, the cells are small with a low *C*_m_, whereas in the periphery they are large with a high *C*_m_ (see Introduction). Along the 1D model, *C*_m_ was varied from 20 to 65 pF according to the following equations.

For *n* = 1, 2,…, 50 (SAN):
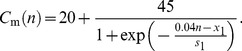
(1)


For *n* = 51, 52,…, 200 (atrial muscle):
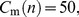
(2)where *n* is the cell number, *x*_1_ is the distance from the SAN centre at which *C*_m_ is midway between 20 and 65 pF and *s*_1_ is the slope factor that describes the steepness of the *C*_m_ gradient.

Previously we determined the relationship between ionic currents and the size of isolated rabbit SAN cells and demonstrated that there is a significant correlation between *C*_m_ and the densities of *I*_Na_, L-type Ca^2+^ current (*I*_Ca,L_), transient and sustained components of the 4-AP sensitive current (*I*_to_ and *I*_sus_), rapid and slow components of the delayed rectifying K^+^ current (*I*_K,r_ and *I*_K,s_), the hyperpolarization-activated current (*I*_f_) and background currents [5], [6]. In the 1D model, conductance (*g*) for these ionic currents was expressed as a linear function of *C*_m_:
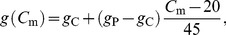
(3)where *g*_C_ and *g*_P_ are the conductances for the central and peripheral SAN models, respectively. The *g*_C_ and *g*_P_ values for each ionic current are listed in Tables S1 and S2. With a *C*_m_ of 20 pF, the SAN model yields a typical central action potential (with d*V*_m_/d*t*_max_ of ∼7 V/s and a MDP of ∼−60 mV). With a *C*_m_ of 65 pF, the SAN model yields a typical peripheral action potential (with d*V*_m_/d*t*_max_ of ∼80 V/s and a MDP of ∼−76 mV).

Combined with a spatial gradient in *C*_m_, this procedure produces regional heterogeneity of the electrophysiological properties in the 1D model (similar to that in earlier works [2], [15]). In the 1D model, when *x*_1_ = +∞, the whole of the SAN string is made of central-type SAN cells (*C*_m_ = 20 pF) and there is an abrupt change in cell type at the SAN-atrial muscle junction. When −∞<*x*_1_<+∞, the cells change from the central-type in the centre of the SAN to the peripheral-type in the periphery of the SAN; the precise distribution is determined by the value of *x*_1_. The slope factor, *s*_1_, was kept constant at 0.2 mm. When *x*_1_ = −∞, the whole of the SAN string is made of peripheral-type SAN cells (*C*_m_ = 65 pF).

In the 1D model, the atrial cells were electrically connected with a coupling conductance (*g*_j_) of 4,000 nS, which is higher than the value obtained experimentally from pairs of rabbit atrial cells (30–635 nS) [12]. The high *g*_j_ was necessary to give a conduction velocity (63 cm/s) similar to that measured experimentally in rabbit atrial muscle (50–80 cm/s) [3]. In most simulations, the cells in the centre of the SAN were electrically connected with a *g*_j_ of 25 nS, which is similar to that measured experimentally (0.6–25 nS) [11]. As explained in the Introduction, it is possible that the *g*_j_ in the periphery of the SAN may be intermediate between that of the centre of the SAN and the atrial muscle. Along the 1D model, *g*_j_ was varied from 25 to 4,000 nS according to the following equations.

For *n = *1, 2,…, 50 (SAN):
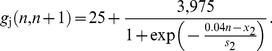
(4)


For *n = *51, 52,…, 99; 101, 102,…,149; 151, 152,…,199 (atrial muscle):
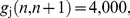
(5)where *x*_2_ is a parameter which determines the distribution of coupling conductance from the centre to the periphery of the SAN. Distance between two neighbouring SAN cells was set to be constant (0.04 mm). In the 1D model, when *x*_2_ = +∞, *g*_j_ in the whole of the SAN string is 25 nS and there is an abrupt change at the SAN-atrial muscle junction. When −∞<*x*_2_<+∞, *g*_j_ changes from 25 to 4,000 nS from the centre to the periphery of the SAN; the precise distribution is determined by the value of *x*_2_. The slope factor, *s*_2_, was kept constant at 0.2 mm. When *x*_2_ = −∞, *g*_j_ in the whole of the SAN string is 4,000 nS. It is not necessary to consider electrical coupling between the three strings of atrial cells, because there are no electrical gradients between the three strings.

The action potential of a particular cell is calculated using the following differential equation:
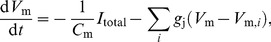
(6)where *V*_m_ is the membrane potential, *I*_total_ is total ionic current and *V*_m,*i*_ is the membrane potential of cells connected to the cell of interest.

### Alternative 1D Model of SAN and Surrounding Atrial Muscle

In order to determine whether the findings are robust, an alternative 1D model was also used. In this case, the model of Zhang et al. [16] was used to compute the action potential in SAN cells and the Oxsoft HEART atrial cell model [17] was used to compute the action potential in atrial cells (Zhang-Oxsoft model). We constructed a 1D string of cells, which consisted of 50 SAN cells (*n* = 1–50) and three parallel strings of 50 atrial cells (*n* = 51–200). As with the SAN model of Kurata et al., the density of ionic currents is a linear function of *C*_m_ in the SAN model of Zhang et al. Along the 1D model, *C*_m_ was again varied. For *n* = 1, 2,…, 50 (SAN), *C*_m_ was calculated according to equation 2. For *n* = 51, 52,…, 200 (atrial muscle), *C*_m_(*n*) = 65 pF. Along the 1D model, *g*_j_ was varied from 25 to 4,000 nS. For *n* = 1, 2,…, 50 (SAN), *g*_j_ was calculated according to equation 4. For *n* = 51, 51,…, 200 (atrial muscle), *g*_j_ was calculated according to equation 5. The action potential was calculated using equation 6.

### 2D Model of SAN and Surrounding Atrial Muscle

A more realistic 2D model of the SAN and surrounding atrial muscle was used to confirm the results obtained with the simple 1D models. We used the 2D model which is similar to our previous 2D model [2]. The SAN is connected to the atrial muscle at the RSARB. However, the rest of the SAN is assumed to be separated from the atrial muscle by connective and fatty tissue. The Kurata et al. SAN cell model and the Lindblad et al. atrial cell model were used in the 2D model. Within the SAN, *C*_m_ was varied from 20 to 65 pF according to the following equation:
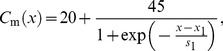
(7)where *x* is the distance from the SAN centre and *x*_1_ is the distance from the SAN centre at which *C*_m_ is midway between 20 and 65 pF (*s*_1_ = 0.2 mm). Within the atrial muscle, *C*_m_ = 50 pF. Distance between two neighbouring cells was 0.04 mm.

Within the SAN, *g*_j_ was varied from 25 to 4,000 nS according to the following equation:
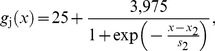
(8)where *x*_2_ is the distance from the SAN centre at which *g*_j_ is midway between 25 and 4,000 nS (*s*_2_ = 0.2 mm). Within the atrial muscle, *g*_j_ = 4,000 nS.

The action potential was calculated using equation 6.

### Validation of 1D and 2D Models - Electrotonic Interaction between the SAN and Atrial Muscle

It is well known that the atrial muscle, because it is more hyperpolarized than the SAN, electrotonically suppresses the pacemaker activity of the SAN. For example, Kirchhoff et al. [18] showed that, on cutting the SAN from the atrial muscle in the rabbit, pacemaker activity became faster. It was checked that the 1D and 2D models behaved in a qualitatively similar manner. In the Kurata-Lindblad 1D model, the cycle length decreased from 314 to 172 ms on removing the atrial muscle (*x*_1_ = 1.2 mm, *x*_2_ = 1.28 mm), similar to that in experiments; in this 1D model, if there was only one string of atrial cells, the change in cycle length on removing the atrial muscle was too small. The inclusion of three strings of atrial cells in the Kurata-Lindblad 1D model can be justified, because at the junction of the SAN with the atrial muscle (at the RSARB), a relatively small number of SAN cells connect to a relatively large number of atrial cells (Fig. 1). In the Zhang-Oxsoft 1D model, the cycle length decreased from 293 to 187 ms on removing the atrial muscle (*x*_1_ = 1.0 mm, *x*_2_ = 1.32 mm). In the 2D model, the cycle length decreased from 301 to 169 ms on removing the atrial muscle (*x*_1_ = 1.3 mm, *x*_2_ = 1.3 mm).

### Safety Factor

The safety factor for conduction (SF) is related to the source-sink relationship and is a measure of the robustness of action potential conduction. Shaw and Rudy [19] defined the SF as the ratio of charge generated to charge consumed during the excitation cycle of a cell. The SF was calculated using the following equation:
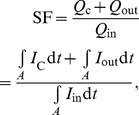
(9)where *Q*_c_ and *Q*_out_ are the sum of the charges that the cell generates for its own depolarization (*Q*_c_) and for the depolarization of downstream cells (*Q*_out_). *Q*_in_ is the sum of the charge that the cell receives from upstream cells. *I*_c_ is the capacitive current of the cell and *I*_in_ and *I*_out_ are axial currents in and out of the cell. The domain of integration, *A*, was defined as the period of time from the moment when the upstroke velocity of the action potential (d*V*_m_/d*t*) reaches 1% of its maximum to the moment when *V*_m_ reaches its maximum [20]. SF>1 indicates that more charge is produced during excitation than the charge required to cause the excitation.

### Computation, Analysis and *in situ* Hybridization

For further details of the Methods, see Supporting Information. Constant values in the models are shown in Tables S1 (Kurata el al. model), S2 (Zhang et al. model), S3 (Lindblad et al. model) and S4 (Oxsoft HEART model). Initial values in the models are shown in Tables S5 (Kurata et al. model), S6 (Zhang et al. model), S7 (Lindblad et al. model) and S8 (Oxsoft HEART model).

## Results

### Relationship between SAN Organisation and SAN Activity

**Figure 2:**
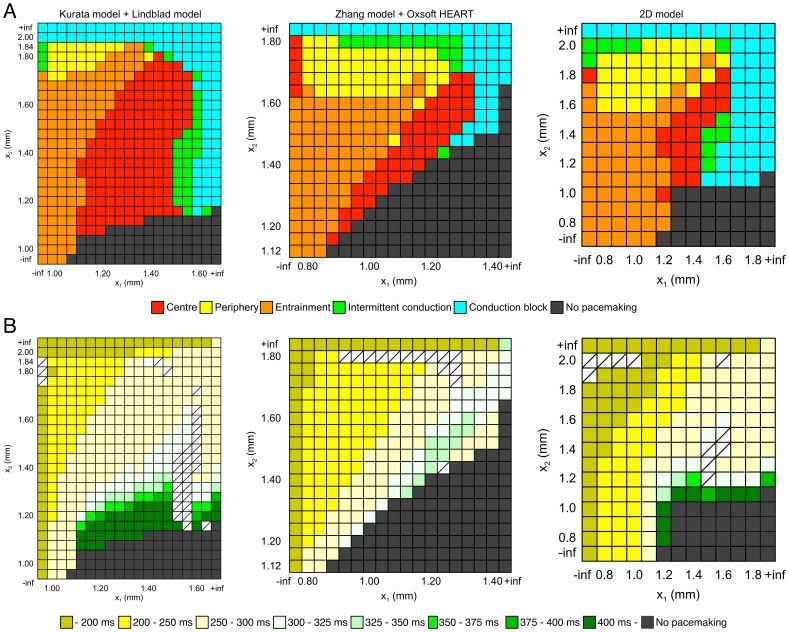
Relationships between organisation of SAN and behaviour and cycle length of SAN in 1D and 2D models. **A**, SAN behaviour. **B**, cycle length. Distribution of *g*_j_ is shown on ordinate and distribution of cell type is shown on abscissa. For each pair of values of *x*_1_ and *x*_2_, behaviour and spontaneous cycle length is colour coded.

Fig. 2 is a summary of the simulation results. This figure shows the relationship of the distributions of cell type and electrical coupling in the SAN - as determined by *x*_1_ (in Eqs. 1 and 7) and *x*_2_ (in Eqs. 4 and 8) - to the electrical behaviour of the SAN (Fig. 2A) and the cycle length of pacemaker activity (Fig. 2B). The abscissa of the graph shows the value of *x*_1_ in the models. This determines the pattern of transition from the central to the peripheral cell type in the SAN. The ordinate of the graph shows the value for *x*_2_ in the models. This determines the pattern of transition in *g*_j_ in the SAN. In this study, depending on the gradients in cell type and electrical coupling in the SAN, we observed six states of pacemaker activity (Fig. 2A):

1. Spontaneous activity originating in the centre of the SAN and successful driving of the atrial muscle; physiological behaviour (*red*)
2. Spontaneous activity originating in the periphery of the SAN and successful driving of the atrial muscle (*yellow*)
3. Entrainment within the SAN defined as firing of all SAN cells at approximately the same time (within 20 ms) and successful driving of the atrial muscle (*orange*)
4. Spontaneous activity in the SAN and intermittent conduction from the SAN to the atrial muscle (*green*)
5. Spontaneous activity in the SAN, but a failure of the SAN to drive the atrial muscle - SAN exit block (*blue*)
6. Complete quiescence: no pacemaking (*grey*).

All the 1D and 2D models show qualitatively similar behaviours (Fig. 2A). Fig. 2A demonstrates that a limited number of combinations of gradients in cell type and electrical coupling in the SAN result in physiological behaviour (the centre of the SAN acting as the leading pacemaker and the SAN driving the atrial muscle; red areas in Fig. 2A). Fig. 2B shows cycle length; in all models, there is a similar pattern: in general, the greater the electrical coupling or the greater the proportion of central cells, the longer the cycle length.

### Gradient either in Electrical Coupling Only or in Cell Type Only is not Sufficient for Physiological Pacemaking and Driving

**Figure 3:**
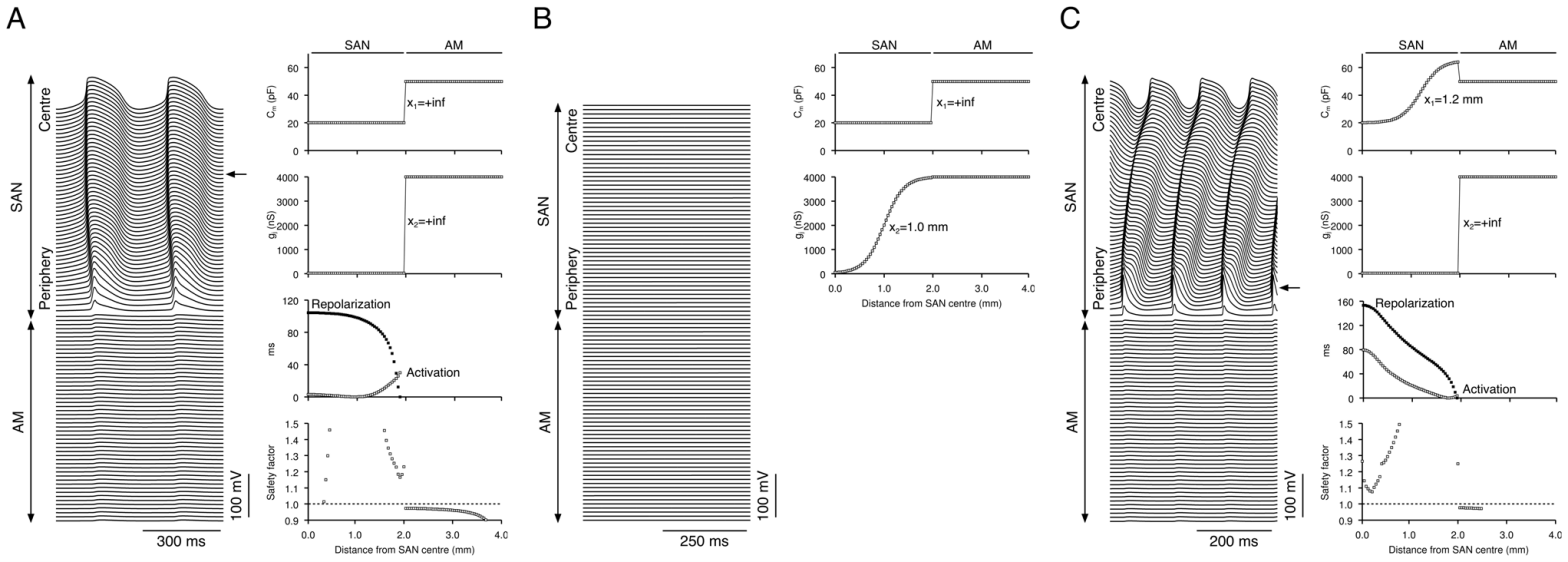
Gradient either in cell type or in electrical coupling only is not sufficient for physiological pacemaking and driving (Kurata-Lindblad 1D model). **A**, conduction failure at SAN-atrial junction (SAN exit block) in a case in which there was no gradient in both cell type and electrical coupling (*x*_1_ = +∞, *x*_2_ = +∞). The whole of the SAN was composed of central-type cells and *g*_j_ = 25 nS throughout the SAN. **B**, electrical quiescence in a case in which a gradient in electrical coupling only was introduced (*x*_1_ = +∞, *x*_2_ = 1.0 mm). The whole of the SAN was composed of central-type cells. g_j_ increased from 25 to 4,000 nS from the centre to the periphery of the SAN. **C**, conduction failure (SAN exit block) in a case in which a gradient in cell type only was introduced (*x*_1_ = 1.2 mm, *x*_2_ = +∞). g_j_ = 25 nS throughout SAN. There was a gradient in cell type from central-type to peripheral-type in the SAN. Left, membrane potential of all cells. Right, *C*_m_ (top), *g*_j_ (second panel), activation and repolarization time (open and filled symbols, respectively; third panel) and safety factor (bottom) along length of model. Arrow, leading pacemaker site.

In Fig. 3A and other similar figures, the calculated membrane potential in all cells is shown on the left. The right panels show *C*_m_ (i.e. cell type) of all cells (top panel), *g*_j_ (i.e. electrical coupling) of all cells (second panel), the activation time (open symbols) and repolarization time (filled symbols) of all cells (third panel), and the SF of all cells (bottom panel).

Fig. 3A shows a simple case with the Kurata-Lindblad 1D model in which there was no gradient in both cell type and electrical coupling in the SAN: a string of 50 identical central-type SAN cells with a *C*_m_ of 20 pF (*x*_1_ = +∞) was coupled to three parallel strings of 50 atrial cells with a *C*_m_ of 50 pF. All the SAN cells were electrically connected with a *g*_j_ of 25 nS (*x*_2_ = +∞). All the atrial cells were coupled with a *g*_j_ of 4,000 nS. In this case (*x*_1_ = +∞, *x*_2_ = +∞), the SAN showed pacemaker activity and the leading pacemaker site was located in the middle of the SAN. However, there was a progressive decrease in the action potential amplitude associated with a progressive slowing of conduction (see plots of activation time in Fig. 3A) towards the periphery of the SAN. Such decremental conduction in the SAN resulted in failure of conduction at the SAN-atrial muscle junction (SAN exit block). SF for conduction decreased progressively from the leading pacemaker site towards the centre and the periphery of the SAN, and SF fell to <1 at the SAN-atrial muscle junction.

Introduction of a gradient in *g*_j_ (by setting *x*_2_ to an appropriate value) without a gradient in cell type (i.e. setting *x*_1_ = +∞) suppressed the pacemaker activity of the SAN, when the whole of the SAN was composed of central-type SAN cells. An example with the Kurata-Lindblad 1D model is shown in Fig. 3B (*x*_2_ = 1.0 mm). This is the consequence of the increase in *g*_j_ in the periphery of the SAN – this enhances the inhibitory electrotonic effect of the more hyperpolarized atrial muscle on the pacemaker activity of the SAN. In the model at least, the whole of the SAN can be composed of peripheral-type cells (*x*_1_ = −∞). With a gradient in electrical coupling, the SAN shows pacemaking and drives the atrial muscle (Fig. 2A). However, in these circumstances, the SAN showed a synchronised activation pattern (entrainment) and the spontaneous cycle length was unphysiologically short (<200 ms; Fig. 2B). These results suggest that a gradient in electrical coupling only is not sufficient for the SAN to show normal and physiological pacemaking and to drive the atrial muscle.

Introduction of a gradient in cell type (i.e. introduction of peripheral-type SAN cells into the periphery of the SAN) tended to enhance the pacemaker activity of the periphery of the SAN. Fig. 3C shows an example with the Kurata-Lindblad 1D model in which the cell type transition was relatively steep (*x*_1_ = 1.2 mm) and all the SAN cells were connected with a *g*_j_ of 25 nS (*x*_2_ = +∞). In this case, the SAN showed stable pacemaker activity (spontaneous cycle length, 150 ms, Fig. 3C) with a cell close to the periphery of the SAN (the ∼45th cell in Fig. 3C as indicated by the arrow) acting as the leading pacemaker site. However, conduction of the action potential was blocked at the SAN-atrial muscle junction, which was associated with a fall of SF to <1 at the junction.

Qualitatively similar results were obtained with the alternative Zhang-Oxsoft 1D model (Fig. S1). These results suggests that a gradient either in electrical coupling only or in cell type only is not sufficient for the SAN to show normal and physiological pacemaking and to drive the atrial muscle.

### Gradients in Electrical Coupling and Cell Type Allow Pacemaking and Driving

In general, a combination of gradients in both cell type and electrical coupling enabled the SAN to show pacemaking and to drive the atrial muscle (Fig. 2). The resultant behaviours can be divided into four types:

#### Intermittent conduction from the SAN to the atrium

When *g*_j_ was relatively low throughout much of the SAN and the SAN was largely composed of central-type cells, the SAN showed pacemaking, but every action potential did not successfully propagate from the SAN into the atrium; an example (Kurata-Lindblad 1D model) is shown in Fig. S2A.

#### Entrainment or electrical synchronisation

When *g*_j_ was high throughout much of the SAN and the SAN was largely composed of peripheral-type cells, all SAN cells fired at approximately the same time because of entrainment or electrical synchronisation; an example (Kurata-Lindblad 1D model) is shown in Fig. S2B. Although the SAN was able to drive the atrial muscle, entrainment of the SAN is not observed physiologically in rabbit SAN-atrial muscle preparations [3].

#### Periphery of the SAN acting as the leading pacemaker

With moderate gradients in both cell type and electrical coupling, the action potential was first initiated in the periphery of the SAN; an example (Kurata-Lindblad 1D model) is shown in Fig. S2C. Once again, although the SAN was able to drive the atrial muscle, this pattern of activation is not normally observed experimentally in rabbit SAN-atrial muscle preparations [3].

#### Physiological behaviour - centre of the SAN acting as the leading pacemaker

**Figure 4:**
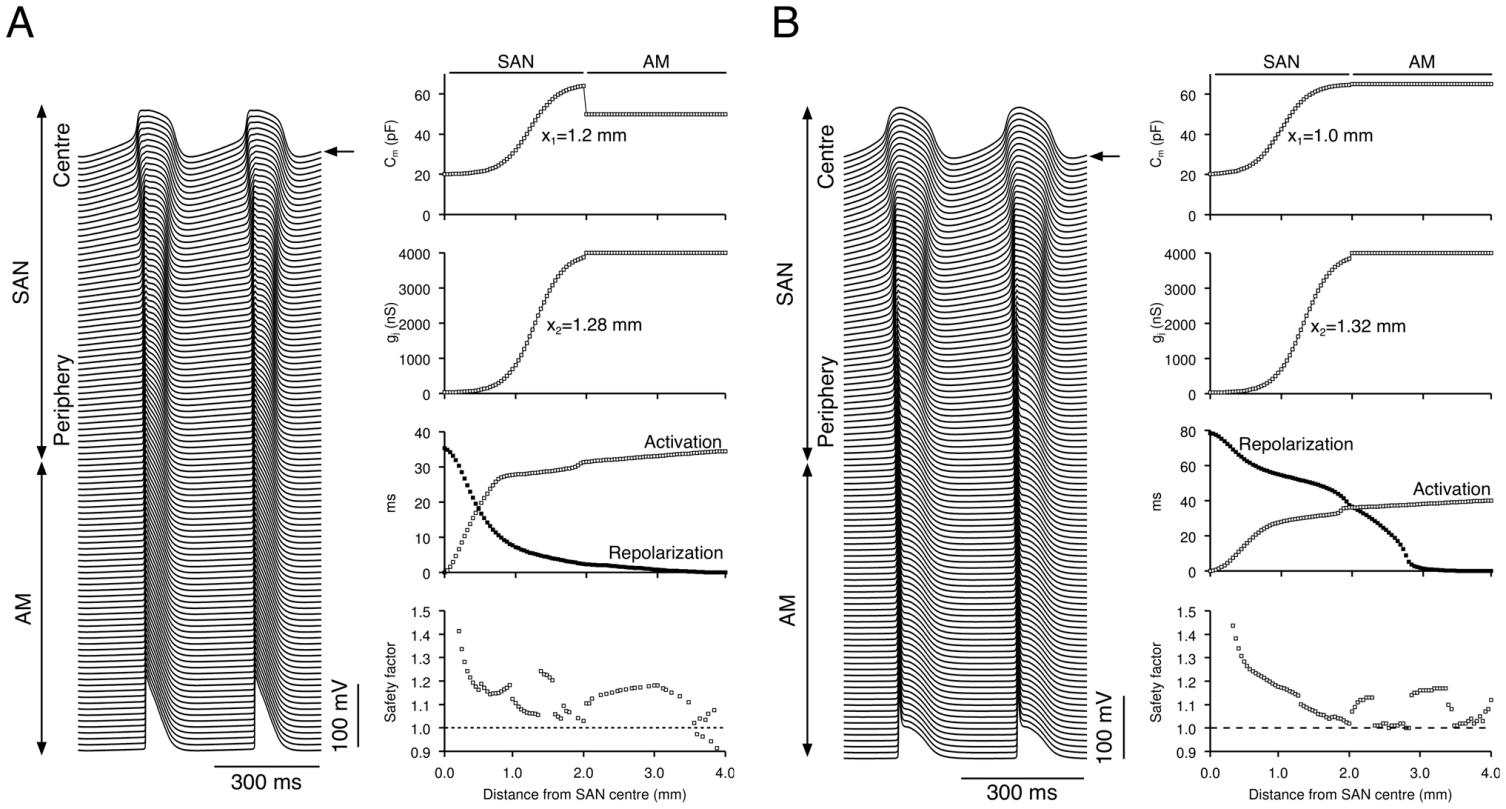
Physiological behaviour in 1D models when there is a gradient both in cell type (from central-type to peripheral-type) and in electrical coupling (*g*_j_ from 25 nS to 4,000 nS). **A**, Kurata-Lindblad 1D model (*x*_1_ = 1.2 mm, *x*_2_ = 1.28 mm). **B**, Zhang-Oxsoft 1D model (*x*_1_ = 1.0 mm, *x*_2_ = 1.32 mm). In both models, spontaneous activity originated in the centre of the SAN and the SAN drove the atrial muscle. Left, membrane potential of all cells. Right, *C*_m_ (top), *g*_j_ (second panel), activation and repolarization time (open and filled symbols, respectively; third panel) and safety factor (bottom) along length of model. Arrow, leading pacemaker site.

Fig. 4 shows examples with the centre of the SAN acting as the leading pacemaker site in the Kudata-Lindblad (Fig. 4A) and Zhang-Oxsoft (Fig. 4B) 1D models. In these examples, there were moderate gradients in both cell type and electrical coupling (*x*_1_ = 1.2 mm, *x*_2_ = 1.28 mm for the Kurata-Lindblad model - Fig. 4A - and *x*_1_ = 1.0 mm, *x*_2_ = 1.32 mm for the Zhang-Oxsoft model - Fig. 4B). The simulation result shows that the action potential originated in the centre of the SAN (the end of the string) with a cycle length of 314 ms with the Kurata-Lindblad model (Fig. 4A) and 293 ms with the Zhang-Oxsoft model (Fig. 4B), and was conducted towards the periphery of the SAN and then into the atrial muscle. SF was >1 throughout the tissue except for a few atrial cells close to the end with the Kurata-Lindblad model. The conduction time from the leading pacemaker site to the SAN-atrial muscle junction was 32 ms with the Kurata-Lindblad model (Fig. 4A) and 36 ms with the Zhang-Oxsoft model (Fig. 4B); this is roughly similar to conduction times observed experimentally [3].

**Figure 5:**
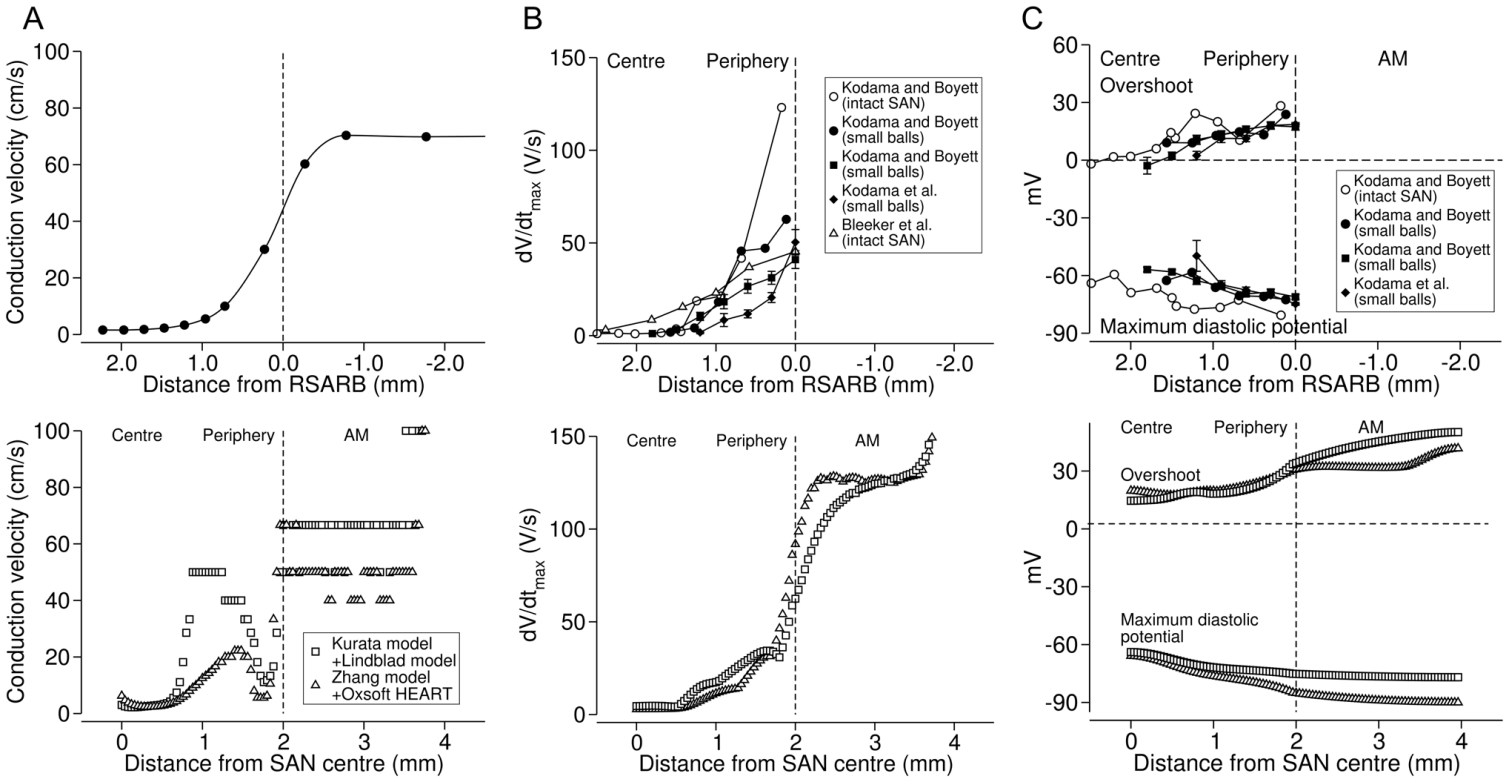
Characteristics of action potentials and conduction velocity in physiological case. **A**, conduction velocity along a line from SAN centre, through SAN periphery to atrial muscle. Top, data from rabbit heart [3]. Bottom, data from Kurata-Lindblad 1D model (*x*_1_ = 1.2 mm, *x*_2_ = 1.24 mm) and Zhang-Oxsoft 1D model (*x*_1_ = 1.0 mm, *x*_2_ = 1.32 mm) shown in Fig. 4 (physiological behaviour). **B** and **C**, maximum upstroke velocity (d*V*_m_/d*t*_max_, **B**) and overshoot and maximum diastolic potential (**C**) of action potentials. Top, data from intact rabbit SAN preparations (Bleeker et al. [22]; Kodama et al. [23]) and small ball-like rabbit SAN preparations (Kodama and Boyett [24]). Bottom, data from Kurata-Lindblad 1D model (*x*_1_ = 1.2 mm, *x*_2_ = 1.24 mm) and Zhang-Oxsoft 1D model (*x*_1_ = 1.0 mm, *x*_2_ = 1.32 mm) shown in Fig. 4 (physiological behaviour). RSARB is a marker for SAN-atrial muscle junction.

Conduction velocity was estimated from the activation time. Conduction velocity was 2–10 cm/s in the centre of the SAN, higher in the periphery, and 40–63 cm/s in the atrial muscle (Fig. 5A, bottom); similar conduction velocities have been observed experimentally (Fig. 5A, top). The repolarization times in Fig. 4A, B show that repolarization proceeded from the atrial muscle to the periphery and then to the centre of the SAN; the same behaviour is observed experimentally [21]. The bottom panels of Fig. 5B,C show d*V*_m_/d*t*_max_, action potential overshoot and MDP plotted versus distance from the SAN centre. From the centre to the periphery of the SAN, the gradients in d*V*_m_/d*t*_max_, action potential overshoot and MDP in the model are similar to those measured experimentally (Fig. 5B,C, top) [22]–[24]. The space constant in the SAN was 430 µm in the Kurata-Lindblad model and 367 µm in the Zhang-Oxsoft model, values roughly similar to space constants measured experimentally in the rabbit heart (205–310 µm) [3].

### Physiological Behaviour in 2D Model

**Figure 6:**
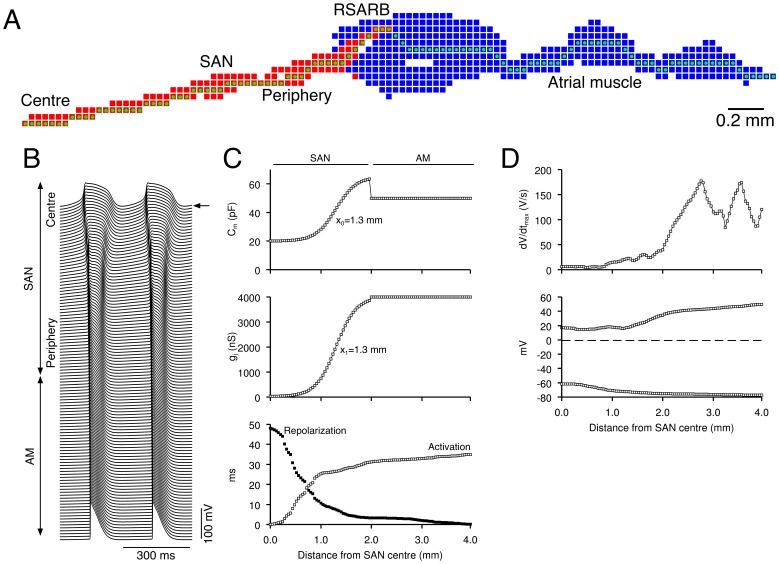
Physiological behaviour in 2D model. **A**, schematic diagram of 2D model. *Red*, SAN cells. *Blue*, atrial muscle cells. **B**, selected action potentials along a line from the SAN centre, through the SAN periphery to the atrial muscle (*green* dots in **A**). **C** and **D**, *C*_m_ (**C**, top), *g*_j_ (**C**, middle), activation and repolarization time (open and filled symbols, respectively; **C**, bottom) and d*V*_m_/d*t*_max_ (**D**, top) and action potential overshoot and maximum diastolic potential (**D**, bottom) along the line from the SAN centre to the atrial muscle. There was a gradient in cell type and electrical coupling (*x*_1_ = 1.3 mm, *x*_2_ = 1.3 mm). Spontaneous activity originated in the centre of the SAN and the SAN drove the atrial muscle. Arrow, leading pacemaker site.

Fig. 6 shows a simulation using the 2D model with gradients in both cell type and g_j_ in which physiological pacemaking and driving of the atrial muscle was observed. Fig. 6A shows the schematic illustration of the 2D model and Fig. 6B shows action potentials along the conduction pathway highlighted by green dots in Fig. 6A. Fig. 6C shows *C*_m_ and *g*_j_ versus distance from the SAN centre along the conduction pathway (*x*_1_ = 1.3 mm, *x*_2_ = 1.3 mm). Fig. 6C also shows the activation and repolarization sequences along the conduction pathway. The leading pacemaker site was in the centre of the SAN, the cycle length was 301 ms, and the activation and repolarization sequences are similar to those observed in animal experiments [3], [21]. Fig. 6D shows d*V*_m_/d*t*_max_ (top), the overshoot and the MDP (bottom) versus distance from the SAN centre. The relationships are comparable to those from experiments (Fig. 5B,C, top). In the 2D model, the SAN tissue was electrically divided into a series of small balls of tissue (Fig. S3) as has been carried out experimentally [23]. In the peripheral ball of tissue, the action potential was larger with a faster upstroke and the pacemaker activity was faster than in the central ball of tissue.

### Importance of Gradient in Na^+^ Conductance in Pacemaking and Driving

**Figure 7:**
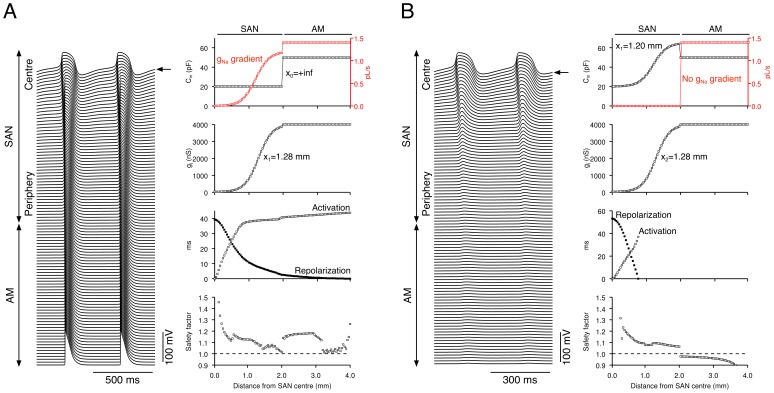
Importance of a gradient in Na^+^ conductance in pacemaking and driving. **A**, the Kurata-Lindblad 1D model with no gradient in cell type (*x*_1_ = +∞) and a gradient in electrical coupling (*x*_2_ = 1.28 mm) normally exhibits pacemaking but no driving of the atrial muscle (see Fig. 2A) - introduction of a gradient in g_Na_ (red trace in top left panel) alone, without changes in other ionic conductances, enables the SAN to drive the atrial muscle. **B**, the Kurata-Lindblad 1D model with appropriate gradients in both cell type and electrical coupling (*x*_1_ = 1.2 mm, *x*_2_ = 1.28 mm) exhibits physiological behaviour (pacemaking and driving of atrial muscle; see Figs. 2A and 4A) - elimination of the gradient in *g*_Na_ (red trace in top left panel) alone from this model, without changes in other ionic conductances, results in a failure to drive the atrial muscle. Left, membrane potential of all cells. Right, *C*_m_ (top), *g*_j_ (second panel), activation and repolarization time (open and filled symbols, respectively; third panel) and safety factor (bottom) along length of model. Arrow, leading pacemaker site.

The results above show that a gradient in cell type as well as a gradient in electrical coupling is essential for normal physiological behaviour. We hypothesised that the key feature of the peripheral cell is the presence of *I*_Na_. We tested this hypothesis in the Kurata-Lindblad 1D model. There was a gradient in electrical coupling (*x*_2_ = 1.28 mm), but not in cell type (*x*_1_ = +∞; all SAN cells were of central type). With this configuration, there was pacemaking but no driving (see Fig. 2A). Introduction of a gradient in Na^+^ channel conductance (*g*_Na_ in Fig. 7A, top right panel), without changes in other ionic conductances, enabled successful driving of the atrial muscle (Fig. 7A). This strongly supports the hypothesis that the essential feature of peripheral cells is attributable to the presence of *I*_Na_.

In order to obtain further evidence to support this hypothesis, we carried out an additional simulation using the Kurata-Lindblad 1D model with gradients in both cell type (*x*_1_ = 1.2 mm) and electrical coupling (*x*_2_ = 1.28 mm) in the SAN. As shown in Fig. 4A, with this configuration, there was pacemaking and successful driving of the atrial muscle. Elimination of the *g*_Na_ gradient in the SAN, without alterations in other ionic conductances, resulted in decremental conduction and failure to drive the atrial muscle (Fig. 7B).

### Pacemaker Shift

The leading pacemaker site and spread of excitation in the SAN are not fixed but dynamically changing [3], [9]. In humans, P wave morphology (indicative of the position of the leading pacemaker site) changes in response to a variety of physiological and pathological situations, including a change in the balance between sympathetic and parasympathetic branches of the autonomic nervous system [25]. In experimental animals, pacemaker shift has been observed with changes in autonomic tone, premature stimulation, overdrive pacing, changes in temperature, changes in extracellular ions and ion channel blockade [3], [24], [26]–[28]. The functional and morphological heterogeneities of the SAN are considered to underlie the shift of the leading pacemaker site in the SAN. In this study, β-adrenergic stimulation (10^−8^ M isoprenaline) caused a shift from the centre to the periphery of the SAN (Fig. S4). Mathematical models of the SAN may provide useful insight into this phenomenon.

## Discussion

In this study, gradient models of the intact SAN and the surrounding atrial muscle were constructed to study the effects of the regional differences in the SAN cells on the initiation and propagation of the action potential. In this study, six types of behaviour were observed depending on the combination of gradients of cell type and electrical coupling in the SAN (Fig. 2). With appropriate gradients of cell type and electrical coupling in the SAN, the behaviour of the SAN (Figs. 4–6) was the same as that seen in experiments in terms of spontaneous cycle length, position of the leading pacemaker site, conduction times and velocities, activation and repolarization sequences, heterogeneity of the action potential, and space constants. Similar findings were obtained with three different models (1D and 2D) and, therefore, the findings are robust.

### Centre of SAN

Fig. 1A, B demonstrates that the centre of the SAN lacks Na_v_1.5 and Cx43– why? Fig. 2A shows that if the SAN is composed of a large number of central cells (*x*_1_>1.04 mm), the SAN will not show pacemaker activity if *g*_j_ is high (4,000 nS) throughout the SAN (i.e. if *x*_2_ = −∞). In this case, the SAN will only show pacemaking, if *g*_j_ is low in the centre of the SAN (i.e. if −∞<*x*_2_<+∞). This demonstrates that electrical coupling in the centre of the SAN needs to be weak in order to protect the central SAN tissue from the inhibitory influence of the atrial muscle. Joyner and van Capelle [1] reached the same conclusion. The atrial muscle has an inhibitory influence on the central SAN tissue, because central SAN tissue is more depolarized than atrial muscle: whereas the MDP in the centre of the SAN is ∼−60 mV, the resting potential of atrial muscle is ∼−80 mV. Central SAN tissue is more depolarized, because it lacks the background inward rectifier K^+^ current, *I*_K1_, responsible for the resting potential of atrial muscle. K_ir_2.1, in part responsible for *I*_K1_, is absent from the human SAN [29].

The sensitivity of central SAN tissue to the hyperpolarizing influence of the atrial muscle is highlighted by a comparison of central SAN tissue with peripheral SAN tissue - peripheral SAN tissue is not as depolarized as the central SAN tissue: the MDP in the periphery of the SAN is ∼−75 mV (∼−60 mV in the centre of the SAN and ∼−80 mV in the atrial muscle). This is thought to be because peripheral SAN tissue has a higher density of *I*_K,r_ than the central tissue [3], [6]. Consequently, it is less affected by the atrial muscle and, as shown in Fig. 2A, if the SAN is composed of a large number of peripheral cells (*x*_1_≤1.04 mm), the SAN will show pacemaker activity if *g*_j_ is high (4,000 nS) throughout the SAN (i.e. if *x*_2_ = −∞). This raises the question why the SAN is not made of peripheral-type cells with the same *g*_j_ as atrial muscle. The action potential of atrial muscle and peripheral SAN tissue is dependent on *I*_Na_, whereas that of central SAN tissue is dependent on *I*_Ca,L_, rather than *I*_Na_ (because of the absence of Na_v_1.5 expression [8], [9], [29]). The action potential in Purkinje fibres, a subsidiary pacemaker tissue, is dependent on *I*_Na_ and consequently it is subject to profound overdrive suppression [30], [31] – following overdrive (rapid stimulation), pacemaker activity can be suppressed for several minutes. During overdrive, intracellular Na^+^ rises as a result of the higher frequency of action potentials (and thus *I*_Na_’s) [31] and this is thought to increase outward Na^+^-K^+^ pump current and, thus, suppress pacemaking [30], [31]. Lethal cardiac stand-still could result if the SAN was also subject to profound overdrive suppression by an ectopic focus (in fact, the SAN exhibits minimal overdrive suppression) [32] and perhaps this is why the action potential at the leading pacemaker site in the SAN (the centre) is dependent on *I*_Ca,L_ and not *I*_Na_. Further studies using more sophisticated mathematical SAN models including detailed intracellular ion dynamics are required to substantiate this theory.

### Periphery of SAN

Fig. 1A, B shows that the periphery of the SAN expresses Na_v_1.5 and Cx43– why? Fig. 2A shows that the SAN cannot only be composed of central SAN cells (i.e. *x*_1_ = +∞). This is true regardless of the distribution of *g*_j_ in the SAN (i.e. for all values of *x*_2_– see Fig. 2A). This is because the *I*_Ca,L_-dependent central-type SAN cells cannot (i) provide enough depolarizing current to stimulate the surrounding atrial muscle and (ii) resist the hyperpolarizing influence of the atrial muscle (Fig. 3A). For the SAN to provide enough depolarizing current to stimulate the surrounding atrial muscle (i.e. drive the atrial muscle) and resist the hyperpolarizing influence of the atrial muscle, there must be *I*_Na_-dependent (Na_v_1.5 expressing) peripheral-type cells in the periphery of the SAN. However, as shown by Fig. 3C this is not the only prerequisite. Fig. 3C shows an example in which peripheral-type cells were present in the periphery of the SAN (*x*_1_ = 1.2 mm), but *g*_j_ was low throughout the SAN (*x*_2_ = +∞). There was stable pacemaker activity, but there was SAN exit block. The low *g*_j_ in the SAN preserved pacemaking, but reduced the coupling current available to initiate atrial firing. Therefore, for the SAN to provide enough depolarizing current to stimulate the surrounding atrial muscle and resist the hyperpolarizing influence of the atrial muscle, there must also be a high *g*_j_ (provided by Cx43) in the periphery of the SAN. In summary, in the periphery of the SAN, both Na_v_1.5 and Cx43 are present (Fig. 1A, B): the Na_v_1.5 provides the necessary depolarizing current to stimulate the surrounding atrial muscle and Cx43 delivers it to the atrial muscle.

Sick sinus syndrome is a dysfunction of the SAN. It is characterised by a bradycardia and can be accompanied by an increase in the SAN conduction time (an increase in the time for the action potential to conduct from the leading pacemaker in the SAN to the surrounding atrial muscle) and SAN exit block (a failure of the action potential to exit from the SAN into the atrial muscle, i.e. a failure of the SAN to drive the atrial muscle) [21]. Familial (i.e. hereditary) sick sinus syndrome has been linked in various families to loss-of-function mutations in Na_v_1.5 [21]. This is paradoxical in that Na_v_1.5 is not expressed in the centre of the SAN (Fig. 1B). This type of the sick sinus syndrome is presumably the result of the role of Na_v_1.5 in the periphery of the SAN being compromised [9], [20].

### Limitations of the Models

Electrical heterogeneity may underlie the robust pacemaker activity of the SAN. The 1D and 2D models used in the present study have heterogeneity only in a direction perpendicular to the crista terminalis. Although, these simplified models may provide a useful tool to test a hypothesis, they are only an approximation of the intact SAN. It is known that there are differences in electrophysiological properties between superior and inferior regions of the SAN and there is also a conduction block zone bordering the SAN [3], [9], and pacemaker shift in response to various interventions including autonomic nerve excitations are partly attributable to these complex electrophysiological heterogeneities [3], [27]. It is an important goal to develop and study more realistic and complex 2D or 3D SAN models surrounded by the atrial muscle of the right atrium and septum (e.g., the 2D model reported by Lang et al. [28]).

## Supporting Information

Figure S1 **Gradient either in cell type or in electrical coupling only is not sufficient for physiological pacemaking and driving (Zhang-Oxsoft 1D model).** **A**, conduction failure at SAN-atrial junction (SAN exit block) in a case in which there is no gradient in both cell type and electrical coupling (*x*_1_ = +∞, *x*_2_ = +∞). The whole of the SAN is composed of central-type cells and *g*_j_ = 25 nS throughout the SAN. **B**, electrical quiescence in a case in which a gradient in electrical coupling only is introduced (*x*_1_ = +∞, *x*_2_ = 1.4 mm). The whole of the SAN is composed of central-type cells. *g*_j_ increases from 25 to 4,000 nS from the centre to the periphery of the SAN. **C**, conduction failure (SAN exit block) in a case in which a gradient in cell type only was introduced (*x*_1_ = 1.0 mm, *x*_2_ = +∞). *g*_j_ = 25 nS throughout the SAN. There is a gradient in cell type from central-type to peripheral-type in the SAN. Left, membrane potential of all cells. Right, *C*_m_ (top), *g*_j_ (second panel), activation and repolarization time (open and filled symbols, respectively; third panel) and safety factor (bottom) along length of model. Arrow, leading pacemaker site.
(TIFF)

Figure S2 **Non-physiological behaviours (Kurata-Lindblad 1D model).** **A**, intermittent conduction from the SAN to the atrial muscle. There is a gradient in cell type and electrical coupling in the periphery of the SAN (*x*_1_ = 1.6 mm, *x*_2_ = 1.8 mm). **B**, electrical synchronisation (entrainment). The SAN is largely composed of peripheral-type cells, and there is a gradient in electrical coupling (*x*_1_ = 1.2 mm, *x*_2_ = 1.6 mm). Spontaneous action potentials in the SAN are synchronised (maximum delay of activation, 10.5 ms) and drive the atrial muscle. Spontaneous cycle length is 261 ms. **C**, spontaneous activity originating from the periphery of the SAN and driving the atrial muscle. There is a moderate gradient in cell type and electrical coupling (*x*_1_ = 1.2 mm, *x*_2_ = 1.8 mm). Left, membrane potential of all cells. Right, *C*_m_ (top), *g*_j_ (second panel), activation and repolarization time (open and filled symbols, respectively; third panel), and safety factor (bottom) along length of model. In **A**, activation and repolarization time and safety factor are shown for two consecutive beats with (left) and without (right) successful propagation to atrial muscle. Arrow, leading pacemaker site.
(TIFF)

Figure S3 **Action potentials and their first derivatives in small balls of SAN tissue from the 2D model (** ***x*** **_1_ = 1.3 mm,** ***x*** **_2_ = 1.3 mm).** **A**, SAN of the 2D model electrically divided into small balls of tissue (A–F; length, 0.28 mm) as has been carried out experimentally by Kodama and Boyett [24]. **B**, spontaneous action potentials (middle) and their first derivatives (bottom) of ball A (periphery), ball C (middle) and ball F (centre).
(TIFF)

Figure S4 **Pacemaker shift in response to sympathetic stimulation.** Effects of isoproterenol (ISO, 1×10^−8^ M) and acetylcholine (ACh, 5×10^−8^ M) on the pacemaker activity was investigated in the Kurata-Lindblad 1D model with a gradient in cell type and electrical coupling (*x*_1_ = 1.2 mm, *x*_2_ = 1.28 mm). Effects of ISO were mimicked by concentration-dependent modification of *I*_f_, *I*_Ca,L_, *I*_K,r_ and *I*_K,s_, as described by Zhang et al. [33], and the effects of ACh were mimicked by concentration-dependent modification of *I*_K,ACh_, *I*_f_ and *I*_Ca,L_ as described by Zhang et al [34]. Before drug application, the model exhibited physiological behaviour: there was spontaneous activity (cycle length, 314 ms) originating in the centre of SAN and driving of the atrial muscle. After application of ISO, there was an acceleration of spontaneous activity (cycle length, 269 ms) and the leading pacemaker site was shifted towards the periphery of the SAN. After application of ACh, there was a deceleration of spontaneous activity (cycle length, 574 ms), but the leading pacemaker site was unchanged.
(TIFF)

Table S1 **Constant values (Kurata et al. model).**
(PDF)

Table S2 **Constant values (Zhang et al. model).**
(PDF)

Table S3 **Constant values (Lindblad et al. model).**
(PDF)

Table S4 **Constant values (Oxsoft HEART model).**
(PDF)

Table S5 **Initial values (Kurata el al. model).**
(PDF)

Table S6 **Initial values (Zhang et al. model).**
(PDF)

Table S7 **Initial values (Lindblad et al. model).**
(PDF)

Table S8 **Initial values (Oxsoft HEART model).**
(PDF)

Text S1 (DOC)
