# Supplementary figures and images for: Importance of Gradients in Membrane Properties and Electrical Coupling in Sinoatrial Node Pacing
Source: PLoS One. 2014 Apr 23;9(4):e94565. doi: 10.1371/journal.pone.0094565 (PMC3997424; doi:10.1371/journal.pone.0094565)

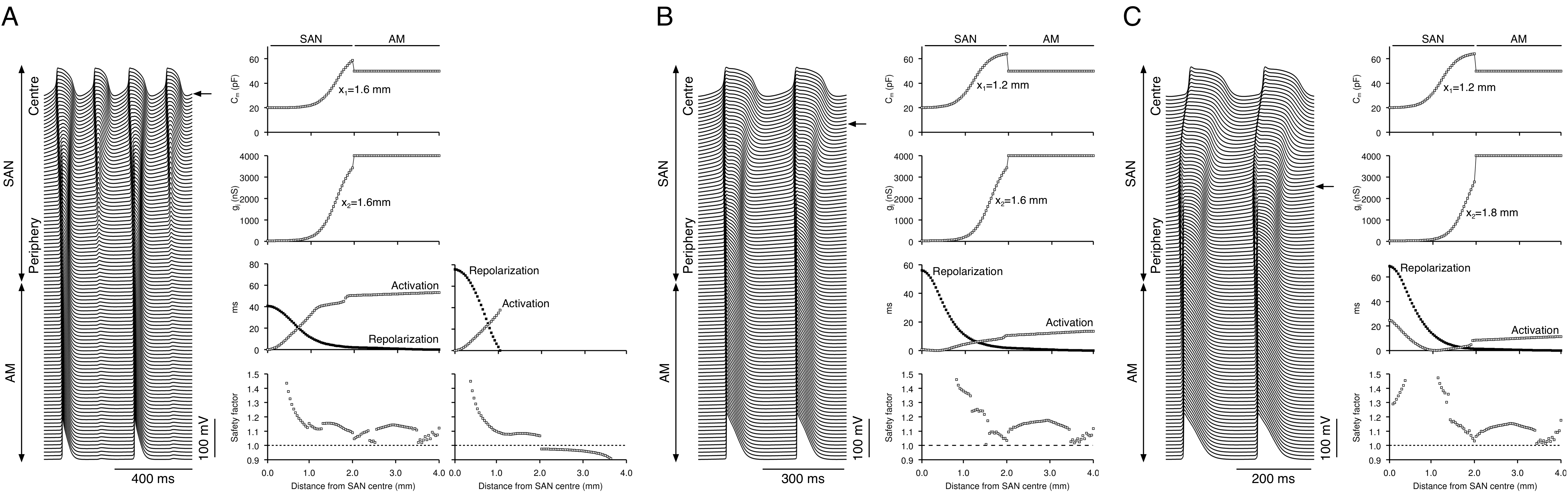

Supplement: Figure S2 — Non-physiological behaviours (Kurata-Lindblad 1D model). A, intermittent conduction from the SAN to the atrial muscle. There is a gradient in cell type and electrical coupling in the periphery of the SAN (x 1 = 1.6 mm, x 2 = 1.8 mm). B, electrical synchronisation (entrainment). The SAN is largely composed of peripheral-type cells, and there is a gradient in electrical coupling (x 1 = 1.2 mm, x 2 = 1.6 mm). Spontaneous action potentials in the SAN are synchronised (maximum delay of activation, 10.5 ms) and drive the atrial muscle. Spontaneous cycle length is 261 ms. C, spontaneous activity originating from the periphery of the SAN and driving the atrial muscle. There is a moderate gradient in cell type and electrical coupling (x 1 = 1.2 mm, x 2 = 1.8 mm). Left, membrane potential of all cells. Right, C m (top), g j (second panel), activation and repolarization time (open and filled symbols, respectively; third panel), and safety factor (bottom) along length of model. In A, activation and repolarization time and safety factor are shown for two consecutive beats with (left) and without (right) successful propagation to atrial muscle. Arrow, leading pacemaker site. (TIFF) [file pone.0094565.s002.tiff]

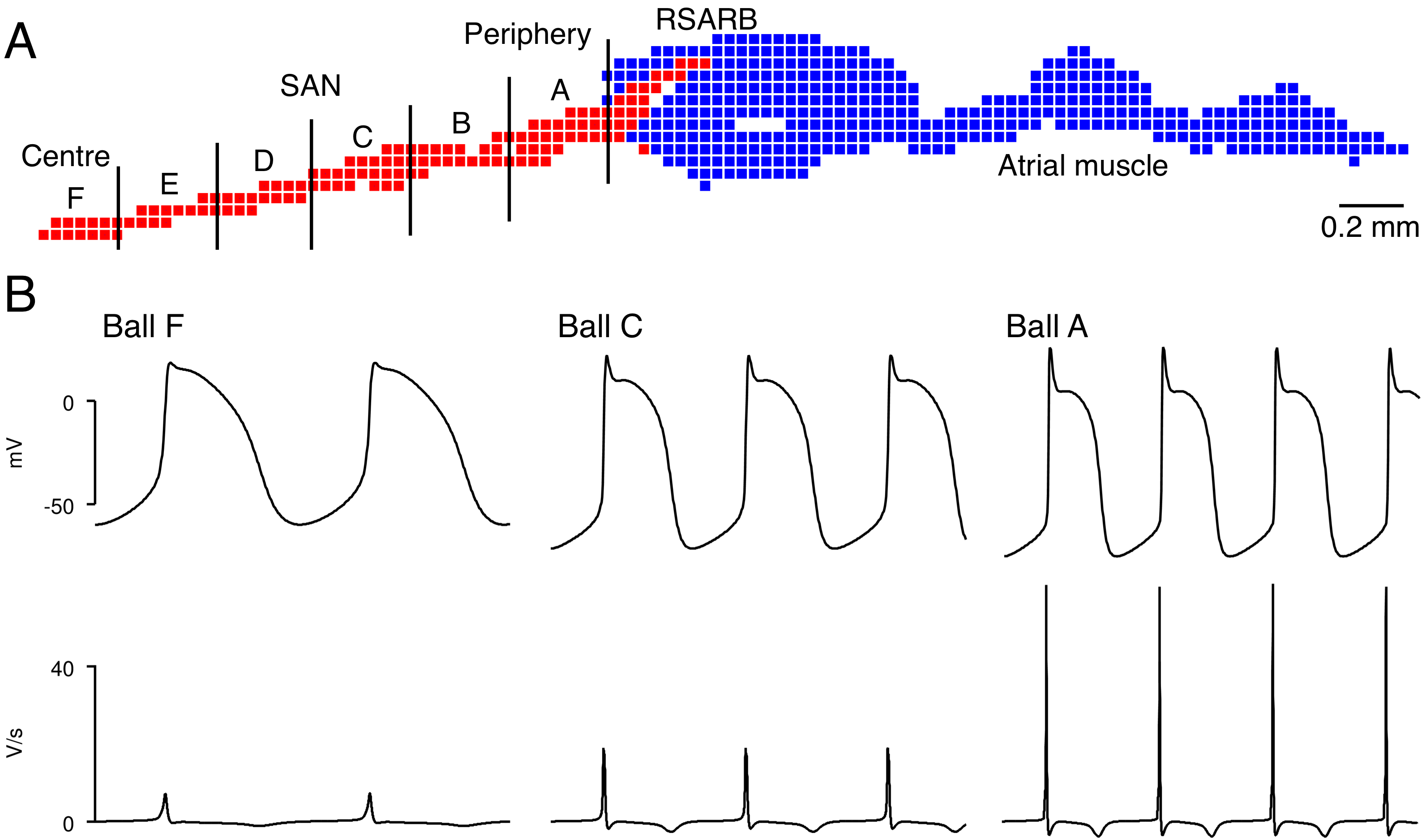

Supplement: Figure S3 — Action potentials and their first derivatives in small balls of SAN tissue from the 2D model ( x 1 = 1.3 mm, x 2 = 1.3 mm). A, SAN of the 2D model electrically divided into small balls of tissue (A–F; length, 0.28 mm) as has been carried out experimentally by Kodama and Boyett [24]. B, spontaneous action potentials (middle) and their first derivatives (bottom) of ball A (periphery), ball C (middle) and ball F (centre). (TIFF) [file pone.0094565.s003.tiff]

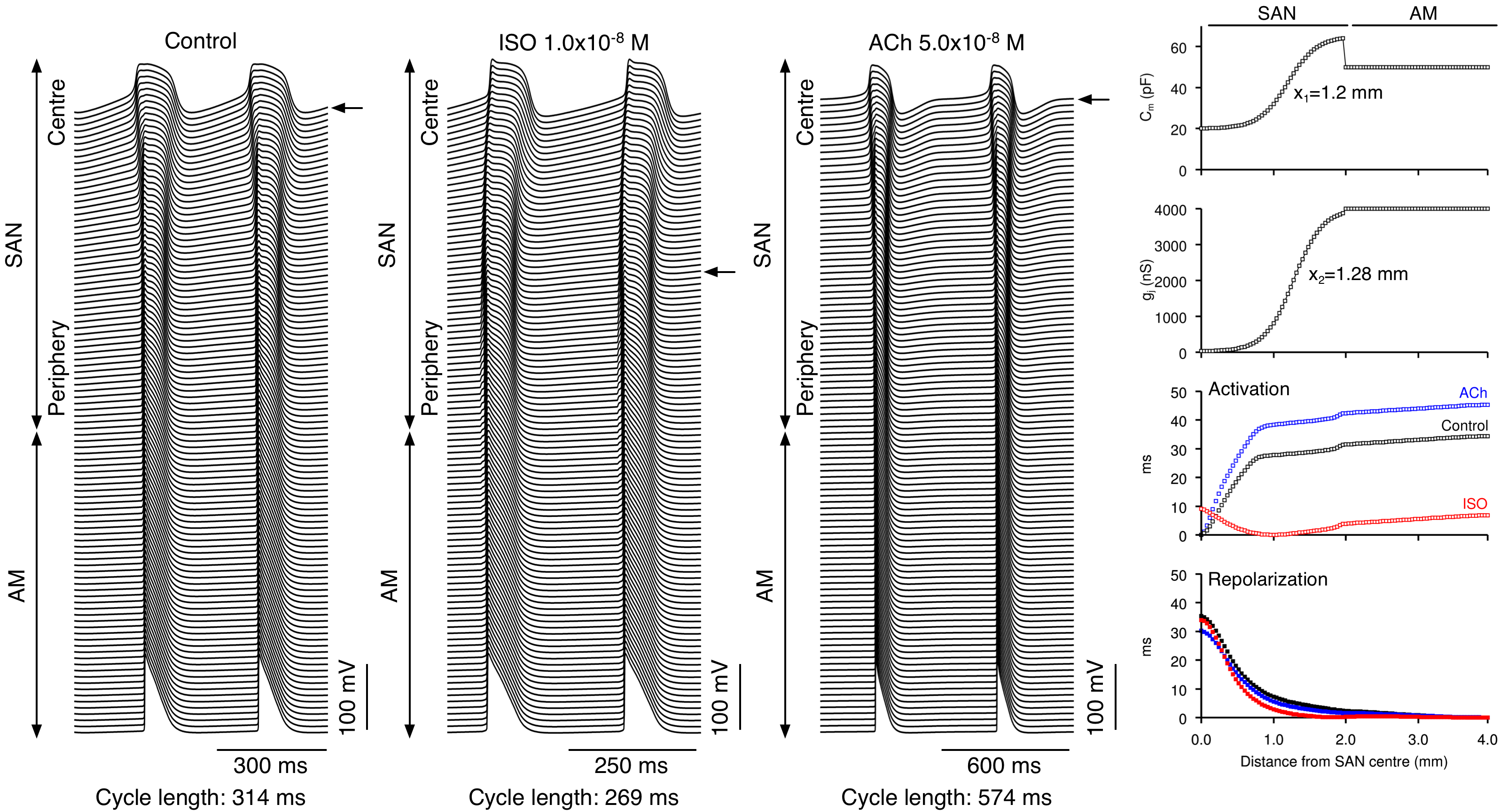

Supplement: Figure S4 — Pacemaker shift in response to sympathetic stimulation. Effects of isoproterenol (ISO, 1×10^−8^ M) and acetylcholine (ACh, 5×10^−8^ M) on the pacemaker activity was investigated in the Kurata-Lindblad 1D model with a gradient in cell type and electrical coupling (x 1 = 1.2 mm, x 2 = 1.28 mm). Effects of ISO were mimicked by concentration-dependent modification of I f, I Ca,L, I K,r and I K,s, as described by Zhang et al. [33], and the effects of ACh were mimicked by concentration-dependent modification of I K,ACh, I f and I Ca,L as described by Zhang et al [34]. Before drug application, the model exhibited physiological behaviour: there was spontaneous activity (cycle length, 314 ms) originating in the centre of SAN and driving of the atrial muscle. After application of ISO, there was an acceleration of spontaneous activity (cycle length, 269 ms) and the leading pacemaker site was shifted towards the periphery of the SAN. After application of ACh, there was a deceleration of spontaneous activity (cycle length, 574 ms), but the leading pacemaker site was unchanged. (TIFF) [file pone.0094565.s004.tiff]
