# Supplementary material for: Importance of Gradients in Membrane Properties and Electrical Coupling in Sinoatrial Node Pacing
Source: PLoS One. 2014 Apr 23;9(4):e94565. doi: 10.1371/journal.pone.0094565 (PMC3997424; doi:10.1371/journal.pone.0094565)
Supplement: Table S1 — Constant values (Kurata et al. model). (PDF) [file pone.0094565.s005.pdf]

	Centre	Periphery
g_{Na} (pL/s)	0.0	1.2
$g_{\mathrm{Ca,L}}$ (nS)	11.6	104.052
$g_{\mathrm{Ca,T}}$ (nS)	9.16	29.77
g_{to} (nS)	3.6	27.261
g_{sus} (nS)	0.4	3.029
$g_{\mathrm{K,r}}$ (nS)	1.352	16.271
$g_{\mathrm{K,s}}$ (nS)	0.518	6.232317
$g_{\mathrm{f,Na}}$ (nS)	0.4189	3.899
$g_{\mathrm{f,K}}$ (nS)	0.6741	6.273
$g_{\mathrm{NaK,max}}$ (pA)	72.0	631.8
k_{NaCa} (nA)	2.5	12.1875
P_{rel} (s^{-1})	1.25×10^3	2.50×10^3
P_{up} (M/s)	0.01	0.02
