# Supplementary material for: Importance of Gradients in Membrane Properties and Electrical Coupling in Sinoatrial Node Pacing
Source: PLoS One. 2014 Apr 23;9(4):e94565. doi: 10.1371/journal.pone.0094565 (PMC3997424; doi:10.1371/journal.pone.0094565)
Supplement: Table S2 — Constant values (Zhang et al. model). (PDF) [file pone.0094565.s006.pdf]

	Centre	Periphery
g_{Na} (pL/s)	0.0	0.37
$g_{\mathrm{Ca,L}}$ (nS)	8.2	65.9
$g_{\mathrm{Ca,T}}$ (nS)	2.1	6.94
g_{to} (nS)	4.905	36.5
g_{sus} (nS)	0.265820	1.14
$g_{\mathrm{K,r}}$ (nS)	0.73820	20.8
$g_{\mathrm{K,s}}$ (nS)	0.3445	10.4
$g_{\mathrm{f,Na}}$ (nS)	0.437	5.5
$g_{\mathrm{f,K}}$ (nS)	0.437	5.5
$g_{\mathrm{b,Na}}$ (nS)	5.8×10^{-2}	0.189
$g_{\mathrm{b_Ca}}$ (nS)	1.32×10^{-2}	4.3×10^{-2}
$g_{\mathrm{b,K}}$ (nS)	2.52×10^{-2}	8.19×10^{-2}
$I_{\mathrm{CaP,max}}$ (pA)	4.2	33.9
$I_{\mathrm{p,max}}$ (nA)	4.78×10^{-2}	0.16
k_{NaCa} (pA)	0.27×10^{-2}	0.88×10^{-2}

Equations for I_{CaP}:

$$I_{\mathrm{CaP}} = I_{\mathrm{CaP,max}} \frac{[\mathrm{Ca}^{2+}]_{\mathrm{i}}}{[\mathrm{Ca}^{2+}]_{\mathrm{i}} + 0.4\mu\mathrm{M}}$$
