# Supplementary material for: Importance of Gradients in Membrane Properties and Electrical Coupling in Sinoatrial Node Pacing
Source: PLoS One. 2014 Apr 23;9(4):e94565. doi: 10.1371/journal.pone.0094565 (PMC3997424; doi:10.1371/journal.pone.0094565)
Supplement: Table S3 — Constant values (Lindblad et al. model). (PDF) [file pone.0094565.s007.pdf]

	Original	Modified
g_{Na}	1.4 (nL/s)	1.4 (pL/s)
$g_{\mathrm{Ca,L}}$ (nS)	4.0	4.0
$g_{\mathrm{Ca,T}}$ (nS)	6.0	6.0
g_{to} (nS)	50.002	35.0
$g_{\mathrm{K,r}}$ (nS)	3.5	7.0
$g_{\mathrm{K,s}}$ (nS)	2.5	5.0
g_{K1} (nS)	5.088	10.0
I_{CaP} (nA)	9.509	9.509
g_{NaCa} (nS)	0.020	0.010
$g_{\mathrm{B,Na}}$ (nS)	0.064	0.064
$g_{\mathrm{B,Ca}}$ (nS)	0.031	0.031
$g_{\mathrm{B,Cl}}$ (nS)	0.120	0.120
