# Supplementary material for: Importance of Gradients in Membrane Properties and Electrical Coupling in Sinoatrial Node Pacing
Source: PLoS One. 2014 Apr 23;9(4):e94565. doi: 10.1371/journal.pone.0094565 (PMC3997424; doi:10.1371/journal.pone.0094565)
Supplement: Table S4 — Constant values (Oxsoft HEART model). (PDF) [file pone.0094565.s008.pdf]

C_{m} (pF)	65
G_{na} (μS)	0.8125
G_{to} (μS)	0.1625
G_{bK} (μS)	2.7625×10^{-3}
G_{K1} (μS)	0.027625
G_{bna} (μS)	1.95×10^{-4}
G_{bCa} (μS)	8.125×10^{-5}
I_{NaKmax} (μS)	0.2275
P_{ca} (nA/mM)	0.08125
k_{NaCa} (nA)	1.625×10^{-4}
Radius (r) (μm)	16.25
Length (l) (μm)	80
V_{cell} (μL)	$10^{-9}\pi \cdot r^2 l$
V_{up}	0.01
V_{rel}	0.1
V_{ecs}	0.4
V_{SRup} (μL)	$V_{\mathrm{cell}} V_{\mathrm{up}}$
V_{i} (μL)	$(1 - V_{\mathrm{ecs}} - V_{\mathrm{up}} - V_{\mathrm{rel}}) \cdot V_{\mathrm{cell}}$
