# Supplementary material for: Importance of Gradients in Membrane Properties and Electrical Coupling in Sinoatrial Node Pacing
Source: PLoS One. 2014 Apr 23;9(4):e94565. doi: 10.1371/journal.pone.0094565 (PMC3997424; doi:10.1371/journal.pone.0094565)
Supplement: Table S5 — Initial values (Kurata el al. model). (PDF) [file pone.0094565.s009.pdf]

	Centre	Periphery
V_m (mV)	-59.0351	-71.1802
m	0.172945	0.0825249
h_1	0.0997523	0.147914
h_2	0.0129199	0.0106793
d_L	5.59853×10^{-4}	7.50960×10^{-5}
f_L	0.570977	0.433570
f_Ca	0.673730	0.443358
d_T	4.25320×10^{-3}	5.64315×10^{-4}
f_T	0.244508	0.234082
q	5.37093×10^{-3}	2.41529×10^{-3}
r	0.489374	0.473830
$p_\mathrm{a,f}$	0.380935	0.590236
$p_\mathrm{a,s}$	0.714068	0.596025
p_i	0.855576	0.922833
x_s	0.0917539	0.0531196
y	0.181862	0.382808
q_a	0.405695	0.0906031
q_i	0.238474	0.309101
$[\mathrm{Na}^+]_\mathrm{i}$ (M)	7.95157×10^{-3}	9.05604×10^{-3}
$[\mathrm{Ca}^{2+}]_\mathrm{i}$ (M)	1.89710×10^{-7}	3.21972×10^{-7}
$[\mathrm{K}^+]_\mathrm{i}$ (M)	0.140162	0.139895
$[\mathrm{Ca}^{2+}]_\mathrm{sub}$ (M)	1.18195×10^{-7}	1.39065×10^{-7}
$[\mathrm{Ca}^{2+}]_\mathrm{rel}$ (M)	3.88524×10^{-4}	2.41973×10^{-4}
$[\mathrm{Ca}^{2+}]_\mathrm{up}$ (M)	2.17832×10^{-3}	5.02177×10^{-3}
f_TC	0.0368051	0.0631722
f_TMC	0.502766	0.653110
f_TMM	0.439108	0.306197
f_CMi	0.0744387	0.123204
f_CMs	0.0375267	0.0570528
f_CQ	0.314701	0.214040
