# Supplementary material for: Importance of Gradients in Membrane Properties and Electrical Coupling in Sinoatrial Node Pacing
Source: PLoS One. 2014 Apr 23;9(4):e94565. doi: 10.1371/journal.pone.0094565 (PMC3997424; doi:10.1371/journal.pone.0094565)
Supplement: Table S6 — Initial values (Zhang et al. model). (PDF) [file pone.0094565.s010.pdf]

	Centre	Periphery
V_{m} (mV)	-57.7016	-80.3264
m	0.187524	0.0472189
h_1	0.102462	0.396606
h_2	0.0135051	0.0232174
d_{L}	2.65806×10^{-3}	6.74224×10^{-5}
f_{L}	0.934503	0.967884
d_{T}	0.0454625	1.70626×10^{-3}
q	0.0298879	9.98983×10^{-3}
r	0.241183	0.245692
$p_{\mathrm{a,f}}$	0.260179	0.309033
$p_{\mathrm{a,s}}$	0.585665	0.357238
p_{i}	0.979192	0.991669
x_{s}	0.156332	0.0745617
y	0.0266851	0.0228544
