# Supplementary material for: Importance of Gradients in Membrane Properties and Electrical Coupling in Sinoatrial Node Pacing
Source: PLoS One. 2014 Apr 23;9(4):e94565. doi: 10.1371/journal.pone.0094565 (PMC3997424; doi:10.1371/journal.pone.0094565)
Supplement: Table S7 — Initial values (Lindblad et al. model). (PDF) [file pone.0094565.s011.pdf]

V_m (mV)	-83.1034
m	2.25434×10^{-3}
h_1	0.986385
h_2	0.952241
d_L	3.93061×10^{-6}
f_L	1.0
d_T	5.25890×10^{-5}
f_T	0.647555
r	5.61573×10^{-6}
s_1	0.908006
s_2	0.330707
s_3	0.617838
p_a	2.64389×10^{-5}
p_i	0.604095
n	5.02689×10^{-3}
$[\mathrm{Ca}^{2+}]_\mathrm{up}$	4.09064×10^{-4}
$[\mathrm{Ca}^{2+}]_\mathrm{rel}$	3.82153×10^{-4}
O_C	0.0181295
O_TnCa	8.71461×10^{-3}
O_TnMgCa	0.155356
O_TnMgMg	0.745338
O_Calse	0.314256
F_1	0.286290
F_2	5.45512×10^{-4}
F_3	0.616048
$[\mathrm{Na}^+]_\mathrm{i}$ (M)	8.49752×10^{-4}
$[\mathrm{Ca}^{2+}]_\mathrm{i}$ (M)	4.38954×10^{-5}
$[\mathrm{K}^+]_\mathrm{i}$ (M)	0.139939
