# Supplementary material for: Importance of Gradients in Membrane Properties and Electrical Coupling in Sinoatrial Node Pacing
Source: PLoS One. 2014 Apr 23;9(4):e94565. doi: 10.1371/journal.pone.0094565 (PMC3997424; doi:10.1371/journal.pone.0094565)
Supplement: Table S8 — Initial values (Oxsoft HEART model). (PDF) [file pone.0094565.s012.pdf]

V_{m} (mV)	-91.6594
m	1.90705×10^{-3}
h	0.992698
d	3.22658×10^{-8}
f	4.15123×10^{-8}
r	0.985791
q	2.43209×10^{-8}
$f_{\mathrm{activator}}$	4.64102×10^{-4}
f_{product}	0.607478
$[\mathrm{Na}^{+}]_{\mathrm{i}}$ (mM)	7.59199
$[\mathrm{Ca}^{2+}]_{\mathrm{i}}$ (mM)	1.07898×10^{-5}
$[\mathrm{K}^{+}]_{\mathrm{i}}$ (mM)	139.968
$[\mathrm{Ca}^{2+}]_{\mathrm{o}}$ (mM)	2.03624
$[\mathrm{Ca}^{2+}]_{\mathrm{up}}$ (mM)	0.554159
$[\mathrm{Ca}^{2+}]_{\mathrm{rel}}$ (mM)	0.551338
$[\mathrm{Ca}^{2+}]_{\mathrm{calmod}}$ (mM)	4.22288×10^{-4}
$[\mathrm{Ca}^{2+}]_{\mathrm{troponin}}$ (mM)	8.04794×10^{-4}
