# Supplementary material for: Importance of Gradients in Membrane Properties and Electrical Coupling in Sinoatrial Node Pacing
Source: PLoS One. 2014 Apr 23;9(4):e94565. doi: 10.1371/journal.pone.0094565 (PMC3997424; doi:10.1371/journal.pone.0094565)
Supplement: Text S1 — (DOC) [file pone.0094565.s013.doc]

**Supporting Information**

**Importance of Gradients in Membrane Properties and
Electrical Coupling in Sinoatrial Node Pacing**

Shin Inada, Henggui Zhang, James O. Tellez, Nitaro Shibata,
Kazuo Nakazawa, Kaichiro Kamiya, Itsuo Kodama, Kazuyuki Mitsui,
Halina Dobrzynski, Mark R. Boyett, Haruo Honjo

**Methods**

**Computation**

All simulations were coded in C++ and MPI (message passing interface) and run on a PC cluster with the Linux operating system. A Runge-Kutta-Fehlberg numerical integration (RKF45) method was used to solve the ordinary differential equations. The time step was 5 µs, which gives a stable solution of the equations and maintains the accuracy of the computation of membrane current and potential. Constant and initial values for the Kurata et al. , Lindblad et al. , Zhang et al. and Oxsoft HEART models are given in Tables S1-S8.

**Analysis**

The activation time (the time interval from the instant of activation at the leading pacemaker site to the instant of activation at the cell of interest) and repolarization time (the time interval from the instant of repolarization at the earliest repolarization site to the instant of repolarization at the cell of interest) of each cell was measured at the level of −30 mV, and these values are plotted versus distance from the SAN centre (*x*) in the figures. Local conduction velocity at a cell of interest, *v*(*n*), was estimated as follows:

$v\left(n\right)=\frac{\Delta x}{t_{\text{act}}\left(n+5\right)-t_{\text{act}}\left(n\right)}$, (1)

where *t*_act_(*n*) and *t*_act_(*n*+5) are the activation times at the cell of interest and at a neighbouring cell, respectively, and ∆*x* is the distance between the two cells (200 µm).

**Space constant**

The space constant in the SAN was measured in the Kurata-Lindblad and Zhang-Oxsoft 1D models. A square wave of hyperpolarizing current (0.5 nA) was injected in the middle of the SAN during electrical diastole. The electrotonic potential in response to the current injection declines with distance from the site of current injection. The decay of the electrotonic potential towards the atrial muscle was fitted by a single exponential function to calculate the space constant.

***In situ* hybridisation**

Male New Zealand white rabbits (1.5-2.5 kg) were killed humanely according to the United Kingdom Animals (Scientific Procedures) Act, 1996, and the SAN was dissected from the isolated hearts and was frozen in liquid N_2_. Cryosections were cut perpendicular to the crista terminalis, approximately at the level of the leading pacemaker site. mRNAs for Na_v_1.5 and Cx43 in the sections were labelled using *in situ* hybridisation as previously described .

**References**

1. Kurata Y, Matsuda H, Hisatome I, Shibamoto T (2008) Regional difference in dynamical property of sinoatrial node pacemaking: role of Na^+^ channel current. Biophys J 95: 951-977.

2. Lindblad DS, Murphey CR, Clark JW, Giles WR (1996) A model of the action potential and underlying membrane currents in a rabbit atrial cell. Am J Physiol 271: H1666-1696.

3. Zhang H, Holden AV, Kodama I, Honjo H, Lei M, et al. (2000) Mathematical models of action potentials in the periphery and center of the rabbit sinoatrial node. Am J Physiol Heart Circ Physiol 279: H397-421.

4. Noble D (1990) Oxsoft HEART. 4.8 ed.

5. Tellez JO, Dobrzynski H, Greener ID, Graham GM, Laing E, et al. (2006) Differential expression of ion channel transcripts in atrial muscle and sinoatrial node in rabbit. Circ Res 99: 1384-1393.
